# Supplementary material for: MetaRibo-Seq measures translation in microbiomes
Source: Nat Commun. 2020 Jun 29;11:3268. doi: 10.1038/s41467-020-17081-z (PMC7324362; doi:10.1038/s41467-020-17081-z)
Supplement: Supplementary file 10 — Supplementary Data 7 [file 41467_2020_17081_MOESM10_ESM.zip › File2/Confidence_High_Taxonomy/92215_out.krona.html]

Javascript must be enabled to view this page.

members
magnitude
magnitudeUnassigned
count
unassigned
taxon
rank

92215\_out

3

3
superkingdom
2

3
phylum
32066

203490
3
class

203491
3
order

203492
3
family

genus
3
848

species
3

SRS023617\_contig\_number\_1047SRS075404\_contig\_number\_1521SRS077633\_contig\_number\_contig-100\_7490.75849
860
